# Hospital Incidence, Treatment, and Outcome of 885 Patients with Thoracoabdominal Aortic Aneurysms Treated in Switzerland over 10 Years—A Secondary Analysis of Swiss DRG Data

**DOI:** 10.3390/jcm12165213

**Published:** 2023-08-10

**Authors:** Kerstin Stoklasa, Anna-Leonie Menges, Benedikt Reutersberg, Lorenz Meuli, Alexander Zimmermann

**Affiliations:** Department of Vascular Surgery, University Hospital Zürich, Raemistrasse 100, 8091 Zurich, Switzerland; kerstin.stoklasa@usz.ch (K.S.); anna-leonie.menges@usz.ch (A.-L.M.); benedikt.reutersberg@usz.ch (B.R.); lorenz.meuli@usz.ch (L.M.)

**Keywords:** thoracic aorta, abdominal aorta, aortic aneurysm, endovascular repair, open repair, epidemiology, diagnosis-related groups

## Abstract

Despite the development of fenestrated and branched endovascular aortic repair (f/bEVAR), the surgical management of thoraco-abdominal aortic aneurysms (TAAAs) remains a major challenge. The aim of this study was to analyse the hospital incidence and hospital mortality of patients treated for TAAAs in Switzerland. Secondary data analysis was performed using nationwide administrative discharge data from 2009–2018. Standardised incidence rates and adjusted mortality rates were calculated. A total of 885 cases were identified (83.2% nonruptured (nrTAAA), 16.8% ruptured (rTAAA)), where 69.3% were male. The hospital incidence rate for nrTAAA was 0.4 per 100,000 women and 0.9 per 100,000 men in 2009, which had doubled for both sexes by 2018. For rTAAA, there was no trend over the years. The most common procedure was f/bEVAR (44.2%), followed by OAR (39.5%), and 9.8% received a hybrid procedure. There was a significant increase in endovascular procedures over time. The all-cause mortality was 7.1% with nrTAAA and 55% with rTAAA. The mortality was lower for rTAAA when f/bEVAR or hybrid procedures were used. A ruptured aneurysm and higher comorbidity were associated with higher hospital mortality. This study demonstrates that the treatment approach has changed significantly over the observed period. The use of f/bEVAR nearly tripled in nrTAAA and doubled in rTAAA during this decade.

## 1. Introduction

Treatment and perioperative management of thoracoabdominal aortic aneurysms (TAAAs, classified as non-ruptured (nrTAAA) and ruptured (rTAAA)) remain a major challenge, not only because of the rarity of this disease (5.9 per 100,000 inhabitants) [1] but also due to the mortality rate ranging from 4.3–14% [2,3,4]. In order to optimise treatment, it is increasingly important to assess epidemiological trends on a regular basis.

Open thoracoabdominal aortic repair (OAR) in complex aortic pathologies is considered the “gold standard” for patients with a life expectancy > 10 years and those with connective tissue disease [5]. However, this is a highly invasive procedure associated with high morbidity and mortality. A large single-center study by Coselli et al. [6] showed a total mortality of 7.5% in 3309 patients with TAAA treated by open surgery (6.2% for nrTAAA vs. 12.2% for rTAAA), while another single-center study by Gombert et al. [7] even showed a total mortality of 20% (16% for nrTAAA vs. 35% for rTAAA).

With the development and introduction of fenestrated and branched endovascular aortic repair (f/bEVAR), aneurysms affecting visceral and renal segments can be treated endovascularly in a single step or staged procedure. As a result, serious complications associated with open thoracoabdominal aortic repair might be avoided, even in symptomatic or ruptured aneurysms.

Large series comparing outcomes after open versus endovascular therapy are rare. A meta-analysis [8] and a systemic review [9] found no significant difference in mortality between open repair and endovascular treatment, despite the higher invasiveness of open surgery. However, reintervention rates were significantly higher in patients who received endovascular treatment. Of note, patients that undergo endovascular therapy are often older and have more comorbidities than patients treated with open repair, potentially biasing direct comparisons.

One possibility to gain greater insight into the care situation of TAAA patients is the use of routinely collected hospital administrative data. This data is primarily used for reimbursement and thereby ensures nationwide coverage. The data allow for detailed epidemiological analyses and allow for comparisons in international contexts where a similar coding for reimbursement is used. For example, Geisbüsch et al. analysed the treatment of patients with TAAA in Germany and showed that there was a significant increase in endovascular treatment in the observed study period. Besides the use of f/bEVAR, treatment in high-volume centers was associated with lower hospital mortality compared with smaller hospitals [10]. For Switzerland, epidemiological data on the treatment of aortic aneurysms is only available for the abdominal segment [11].

The aim of this study was to better understand the epidemiology of TAAA in Switzerland, including treatment incidence, treatment modality, and hospital mortality.

## 2. Methods and Statistics

### 2.1. Dataset

This is a secondary data analysis of case-related hospital discharge data from the Swiss Federal Statistics Office (SFSO) from the period 2009–2018. All hospitals, birthing centers, and specialised medical facilities in Switzerland are obliged to report all admissions to the SFSO annually. The SFSO collects sociodemographic variables, such as age, sex, type of admission, and discharge data (including hospital mortality) for each case. Admission information include pre-hospital location and admission type. Further information includes diagnosis codes, up to 100 procedure codes, insurance class, total length of stay (days), and length of stay in the intensive care unit (ICU). Diagnoses are recorded using the 10th revision of the International Classification of Diseases (ICD-10), while procedures are recorded using the Swiss classification of surgical interventions (CHOP [12]).

Because of regulations used to protect personal data, patients cannot be identified by unique identifiers and institution numbers are encoded. Therefore, the dataset does not allow for identifications of readmissions for previously treated patients and the linkage of follow-up information after discharge is not possible. The institutions are classified into five levels of care, and information on the destination after discharge is provided in the hospital discharge data.

The local ethics committee waived ethical approval for this analysis of fully anonymised data (BASEC-Nr. Req-2021-01010). This study followed the reporting guidelines of the STROBE statement.

### 2.2. Inclusion and Exclusion Criteria

This study included all cases from the years 2009–2018, where a diagnosis of nrTAAA (ICD-10: I71.6) or rTAAA (ICD-10: I71.5) was coded as either the primary or any secondary diagnosis. Open repair cases were identified using CHOP codes 38.34 “resection of aorta with anastomosis”, 38.45 “resection and replacement of thoracic vessel with anastomosis”, 38.44 “resection of abdominal aorta with anastomosis”, and 38.45 “resection and replacement of abdominal aorta with anastomosis”, while endovascular repair cases were identified using CHOP codes 39.71 “endovascular implantation of stentgraft into abdominal aorta”, 39.73 “endovascular implantation of stentgraft into thoracic aorta”, and 39.79 “endovascular correction of aneurysm other vessel”. Cases that had both open and endovascular codes were classified as hybrid treatment. Cases with both I71.5 and I71.6 codes were excluded. To avoid duplicates, all cases that had been transferred to another hospital without surgical treatment; double entries; and cases from rehabilitation, geriatric, or psychiatric clinics without surgical treatment were excluded. In addition, implausible cases where rTAAA was coded as a primary diagnosis without surgical treatment but the length of hospital stay was more than three days were excluded. Likewise, all cases in which rTAAA was coded as a secondary diagnosis without surgical treatment for the rTAAA were excluded.

### 2.3. Statistical Analysis

For the descriptive analyses, the mean and standard deviation (SD) were reported for approximately normally distributed continuous variables. For variables with a skewed distribution, the median and interquartile range (IQR) were calculated. For categorical variables, frequency and percentage were given. Normality was visually inspected using histograms. Factor variables were compared using the chi-squared test, while continuous variables using ANOVA if normally distributed or the Kruskal–Wallis rank test otherwise. Comorbidities were summarised using a sum score of the weighted Elixhauser ICD-10 diagnosis groups according to van Walraven [13]. This is a method where the presence of ICD-10 codes for each comorbidity category is evaluated for each case and summed up using a weighting system. The weighting algorithm by van Walraven was developed based on the association between comorbidity and death. The van Walraven score was calculated using the “comorbidity” R package version, 0.5.3. Further, the dataset was scanned separately for the most relevant comorbidities. Definitions were used as established in the Elixhauser comorbidity score for arterial hypertension and chronic pulmonary disease and as in the Charlson comorbidity score for peripheral vascular disease, diabetes, and renal disease. The dataset was further scanned for additional procedures, including the transfusion of blood products, spinal fluid drainage, neuromonitoring, use of a heart–lung machine, and the use of intraoperative blood salvage devices, as well as for complications, including bowl-resection, hemodialysis, acute paraplegia, lower limb fasciotomy, and major limb amputation (at or proximal to the ankle).

To analyse the association between treatment modality and hospital mortality, a multivariable logistic regression model was built for the surgically treated cohort. The variables age (continuous), sex (factor), van Walraven comorbidity score (continuous), type of treatment (OAR versus f/bEVAR versus hybrid repair), diagnosis (ruptured versus non-ruptured), insurance class (private versus general), type of admission (direct admission versus hospital transfer), hospital level (university versus non-university), and period of treatment (2009–2013 versus 2014–2018) were included to adjust for potential confounding. The model was reported using odds ratios (ORs) with the corresponding 95% confidence interval (95% CI) and *p*-values.

The data structure did not allow for missing data. All analyses were performed using R version 4.2.3 on macOS 12.5.1 (Apple, Cupertino, CA, USA). All *p*-values were two-sided with an alpha level of 5%.

## 3. Results

**Figure 1:**
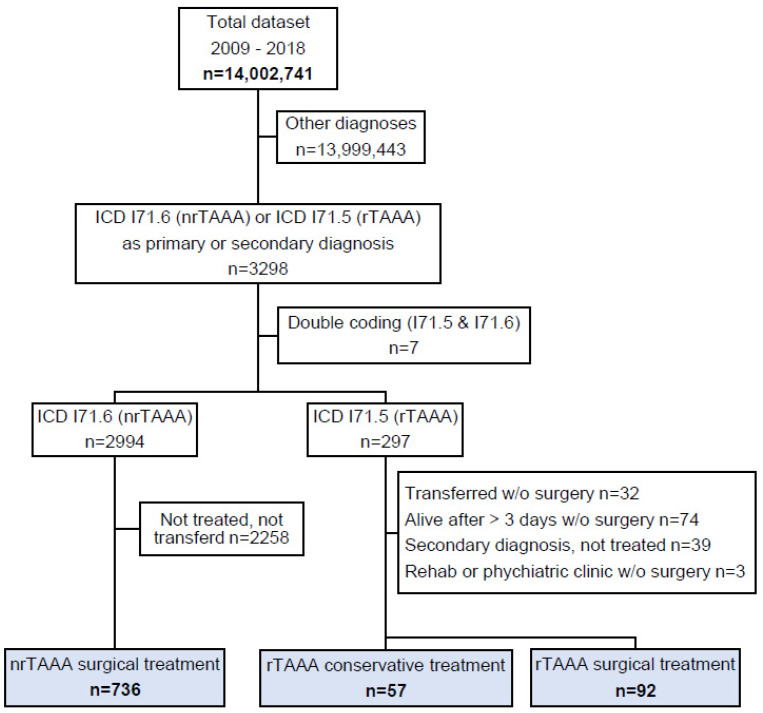
Patient flow. The total dataset contained all hospitalisations in the Swiss population in the years from 2009–2018. ICD: International Classification of Diseases (version 10); nrTAAA: non-ruptured thoracoabdominal aortic aneurysm; rAAA: ruptured thoracoabdominal aortic aneurysm.

From 2009–2018, a total of 3298 cases were hospitalised in Switzerland with nrTAAA or rTAAA as their main or secondary diagnosis. A final study cohort of 885 cases was identified after excluding 2258 cases with nrTAAA as a diagnosis but no coded surgical procedure, 32 cases with rTAAA transferred from another hospital without treatment, and 116 potential duplicates (Figure 1). Of the 885 cases, 736 (83.2%) received treatment for nrTAAA, 92 (10.4%) were treated for rTAAA, and the remaining 57 (6.4%) cases had rTAAA but did not undergo surgical treatment. Table 1 and Table 2 summarise the baseline characteristics of the nrTAAA and rTAAA cohorts.

### 3.1. Epidemiology: Hospital Incidence of TAAA

**Figure 2:**
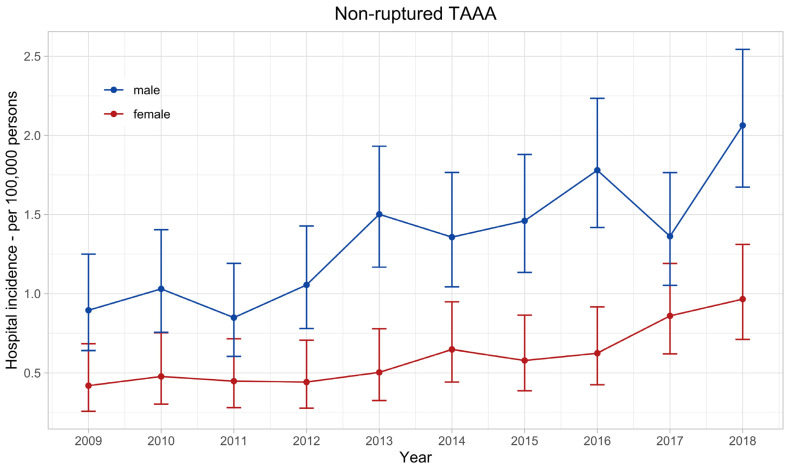
Hospital incidence of non-ruptured thoracoabdominal aortic aneurysm. The standardised incidence rates are presented with 95% confidence intervals for each year and stratified by sex.

The age-standardised annual hospital incidence of nrTAAA doubled in the observed decade in both sexes (Figure 2), from 0.42 (95% confidence interval: 0.26–0.68) per 100,000 women and 0.9 (0.64–1.25) per 100,000 men in 2009–0.97 (0.71–1.31) and 2.06 (1.67–2.54), respectively. The mean annual hospital incidence of nrTAAA in men was 1.35 (1.24–1.47) and significantly higher compared with women, with a mean annual hospital incidence of 0.60 (0.53–0.69), *p* < 0.001.

**Figure 3:**
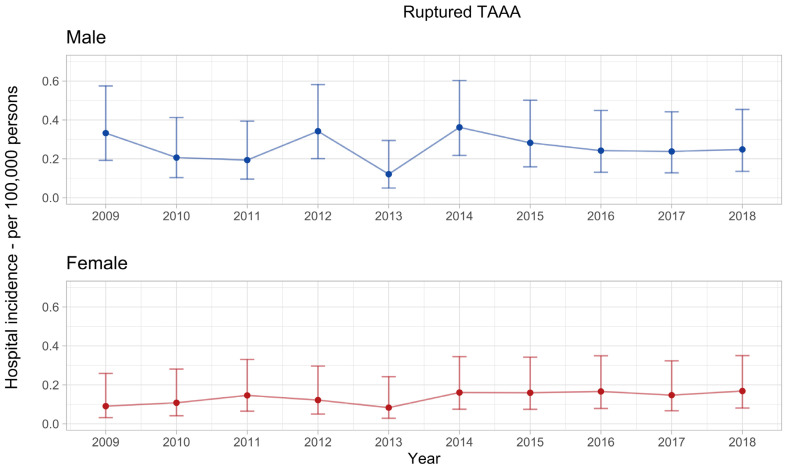
Hospital incidence of ruptured thoracoabdominal aortic aneurysm. The standardised incidence rates are presented with 95% confidence intervals for each year and stratified by sex. The overall incidence rate in men was 0.26 (0.21–0.31) and significantly higher compared with women, with an overall incidence rate of 0.14 (0.10–0.18), *p* < 0.001.

The hospital incidence of rTAAA had no apparent trend in the observed decade. The mean annual hospital incidence was 0.26 (0.21–0.31) per 100,000 men and significantly higher compared with women, with a mean annual hospital incidence of 0.14 (0.10–0.18) per 100,000 women, *p* < 0.001 (Figure 3).

Most of the 736 elective nrTAAA cases were treated in large hospitals, with 83.6% in university hospitals and 11.4% in other large hospitals. The remaining 5% of cases were treated in small hospitals (regional hospitals and specialty clinics) (Table 1). Similar figures were observed in the treatment of rTAAA, with 91.9% of cases treated in either university hospitals (64.4%) or large hospitals (27.5%). Of note, 59% of the surgically treated cases with rTAAA were transferred prior to treatment from other acute care hospitals (Table 2).

### 3.2. Time Trends in Treatment Modality

**Figure 4:**
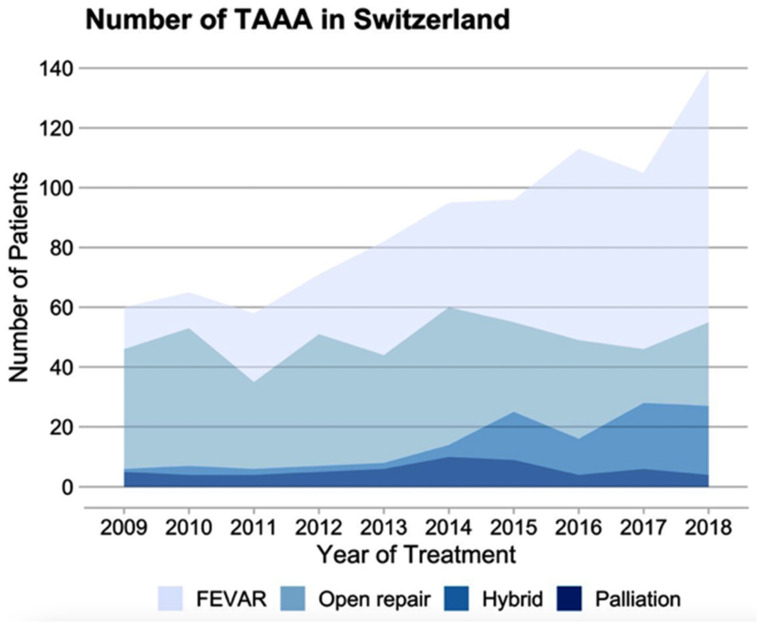
Time trends for TAAA treatment. Absolute number of cases treated for nrTAAA or rTAAA between 2009 and 2018 in Switzerland. FEVAR: fenestrated or branched endovascular aortic repair.

Of all the cases treated for nrTAAA, 348 received f/bEVAR, 309 received OAR, and 79 cases were treated with a hybrid repair (Table 1). For rTAAA, 43 cases received f/bEVAR, 41 received OAR, 8 cases were treated with a hybrid repair, and 57 cases received palliative care (Table 2). Figure 4 demonstrates that endovascular and hybrid procedures consistently rose over the decade. In 2009, 21.7% of nrTAAA cases underwent f/bEVAR, while this percentage significantly increased to 61% in 2018. Similar changes were observed in the treatment of rTAAA, with 28.6% receiving f/bEVAR in 2009 and 58.8% in 2018. The proportion and absolute number of open repairs performed decreased over the observed decade.

### 3.3. Hospital Mortality

The overall hospital mortality was 7.1% for nrTAAA (Table 3). The hospital mortality rates were similar between different treatment modalities with 6.6% (23/348) for f/bEVAR, 7.4% (23/309) for OAR, and 7.6% (6/79) for hybrid procedures. The comparative subgroup analysis of 2009–2013 and 2014–2018 did not show a significant difference in mortality over time (*p* = 0.915, Table S1).

**Figure 5:**
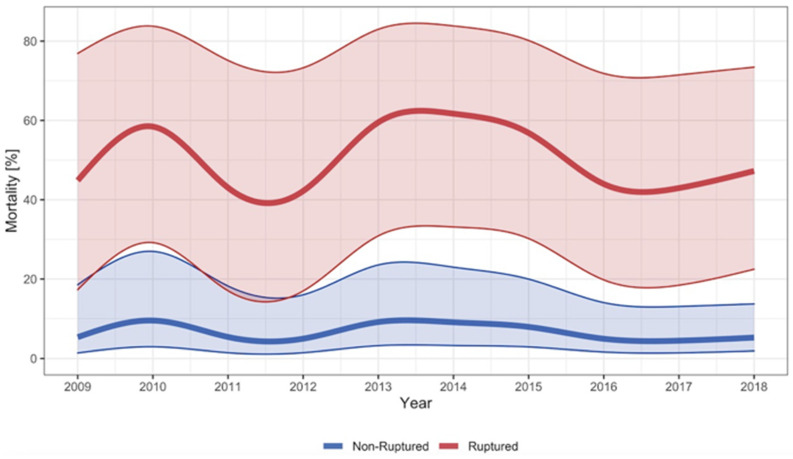
Hospital mortality for TAAA. Smoothed and adjusted hospital mortality of cases surgically treated for non-ruptured TAAA and hospitalised for ruptured TAAA (surgery and palliative care) in Switzerland between 2009 and 2018. The shadowed blue and red area around the curves indicate the 95% confidence interval. Mortality rates were adjusted for age, sex, year of treatment, and van Walraven score.

The overall hospital mortality for rTAAA was 55%. For surgically treated cases with rTAAA, the hospital mortality was 29%, and for palliative care cases, the hospital mortality was 97%. The mortality was similar for f/bEVAR (28%, *n* = 12/43) and OAR (34%, *n* = 14/42) and lower for the few cases treated with a hybrid procedure (13%, *n* = 1/8). The mortality rates were stable in the observed decade (see Figure 5). This was confirmed in the subgroup analysis that compared the period 2009–2013 with 2014–2018, where no significant difference in the mortality rate was observed, *p* = 0.708 (see Table S2).

**Figure 6:**
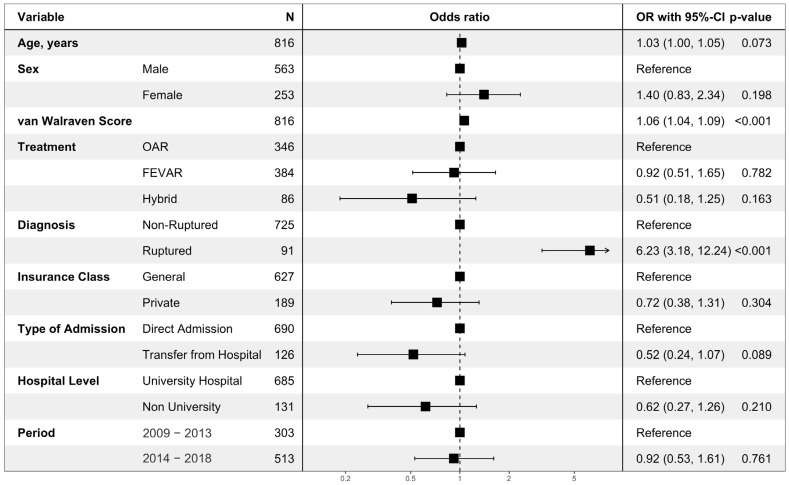
Multivariable regression analysis on hospital mortality. The multivariable logistic regression model on all cases surgically treated for nrTAAA (*n* = 736) or rTAAA (*n* = 92). Cases with rTAAA without surgical treatment (*n* = 57) were excluded. A total of 79 events were observed. Data was complete. Odds ratios are presented with corresponding 95% confidence intervals and *p*-values. OAR: open aortic repair; FEVAR: fenestrated or branched endovascular aortic repair.

The multivariable analysis on hospital mortality showed that a ruptured aneurysm (odds ratio (OR) 6.23, 95% confidence interval: 3.18–12.24, *p* < 0.001) and higher van Walraven Score (OR 1.06 per point (1.04–1.09, *p* < 0.001)) were significantly associated with hospital mortality. There was a tendency towards lower mortality when interhospital transport was performed preoperatively (OR 0.52 (0.24–1.07, *p* = 0.089)). There was no statistically significant difference in mortality between the first 5 years (2009–2013) and the last 5 years (2014–2018) (OR 0.92 (0.53–1.61, *p* = 0.761); see Figure 6).

### 3.4. Secondary Outcomes: Complications

Table 3 summarises the management and treatment outcomes for cases treated for nrTAAA. The use of cerebrospinal fluid (CSF) drainage and motor evoked potentials (MEPs) monitoring was higher for OAR compared with f/bEVAR. In comparison with the years 2009–2013, the use of CSF significantly increased between 2014 and 2018 (*p* < 0.001, Table S1). The rate of acute paraplegia was twice as high in patients with OAR (5.2%) compared with f/bEVAR (2.6%). An HLM was used in 70.2% of cases for OAR and in 62% for hybrid procedures. Transfusion rates were higher in OAR compared with f/bEVAR for packed red blood cells, fresh frozen plasma, and platelet concentrates and were transfused significantly more frequently from 2014–2018 than in the previous years (*p* < 0.001, Table S1). Postoperative dialysis requirement for renal insufficiency occurred more frequently after hybrid procedures (20.3%) than after OAR (8.7%) or f/bEVAR (6.0%). Rare complications in nrTAAA cases included large or small bowel resection, which occurred in 2.5% of cases after hybrid repair, in 4.2% of cases after OAR, and in 1.7% of cases after f/bEVAR.

Table 4 summarises the management and treatment outcomes for cases treated for rTAAA. The use of CSF drainage (*n* = 2/97) and MEPs monitoring (*n* = 3/97) was very low in the rTAAA cohort and there was no difference in use over the years. HLM was used in 66% of cases with OAR for rTAAA cases and in 38% of cases for hybrid procedures. There was also a significantly higher amount of packed red blood cell transfusions in the rTAAA group in the later period (2014–2018) compared with the earlier period (2009–2013) (*p* < 0.001, Table S2). The diagnosis of acute paraplegia was roughly twice as high after treatment for rTAAA than after elective treatment for nrTAAA and twice as high after OAR (10%) than after f/bEVAR (5%). Likewise, the use of CVVHD was almost twice as high in cases after rTAAA compared with nrTAAA but similar for f/bEVAR (14%) and OAR (12%).

## 4. Discussion

This was the first study to show the nationwide hospital incidence of TAAA treatment in Switzerland. Within its limitations, this case-related hospital discharge data can be used to estimate the actual incidence and treatment outcomes of patients treated for TAAA. This specifically applies for OAR where the entire pathology is generally treated within a single procedure. For f/bEVAR, where patients are often treated in two or more stages, the reported case incidence might be substantially higher than the actual patient incidence.

### 4.1. Epidemiology

The annual hospital incidence rate in the observed decade for nrTAAA was 0.6 per 100,000 women and 1.35 per 100,000 men. For rTAAA, these rates were 0.14 per 100,000 women and 0.26 per 100,000 men. Between 2009 and 2018, there was a doubling of the incidence of nrTAAA in both men and women, while the incidence rates for rTAAA were stable. The observed incidence rates for TAAA were comparable to data from Germany [10]. In a comparable study, Geisbüsch et al. showed a threefold increase in incidence for Germany between 2005 and 2014 [10]. Besides the repeating counts of staged endovascular procedures, this increase might be attributed to the increasing availability and lower invasiveness of endovascular techniques, which expand the spectrum of patients eligible for elective treatment [14]. Furthermore, the increased use of modern radiological imaging such as CT and MRI to detect and grade malignancies in the older population may have led to an increase in incidental findings of TAAA [15].

### 4.2. Hospital Mortality

Increasing experience in open surgical treatment and the development and broad use of endovascular therapy have led to significant improvements in the treatment of TAAA [11,15]. These advances resulted in a more individualised, patient-specific therapy based on comorbidities, aneurysm morphology, and the presence of connective tissue disease [5,16]. Geisbüsch et al. reported decreasing age, sex, and comorbidities standardised mortality rates for the treatment of nrTAAA from 21% in 2005 to 11% in 2014 [10]. Lower mortality rates for nrTAAA in Germany were associated with endovascular treatment and higher hospital case volume [10]. In Switzerland, the comparably adjusted mortality rates were significantly lower at 7% (OAR 7.4%, f/bEVAR 6.6%) and stable in the observed decade. Despite the wider use of endovascular therapy in Switzerland and the increasing number of treatments for nrTAAA, neither mortality nor rupture rates decreased.

The observed mortality rates in Switzerland were consistent with a systematic review, reporting similar mortality rates [9]. On the other hand, in a large retrospective cohort study, the mortality rate for the endovascular treatment of TAAA was only 4.9% [17]. In contrast with these excellent results, a recently published large cohort study of a high-volume center for open aortic surgery showed mortality rates of 16% in nrTAAA and 35% in patients with symptomatic TAAA, excluding ruptured cases [7]. In Switzerland, the mortality rate for surgically treated rTAAA was at around 30% (34.1% for OAR and 27.9% for f/bEVAR).

The multivariable analysis of this study found that aneurysm rupture and a higher van Walraven score were associated with higher mortality. A population-based Canadian study conducted from 2006–2017 found that older age, chronic kidney disease, congestive heart failure, and urgent surgery were associated with higher mortality and complication rates. Of note, the type of surgery was not associated with mortality in either our study or the population-based Canadian study [18]. However, endovascular repair was associated with lower mortality in the population-based study from Germany [10].

### 4.3. Complications

Complications after surgical treatment of TAAA are potentially serious. Besides a haemorrhage with the need for mass transfusion, ischaemic complications can result in renal failure; bowel ischaemia; and most dramatically, spinal cord ischaemia with acute paraplegia. To keep the number of these complications as low as possible, the use of a heart–lung machine, a CSF drainage, and extensive transfusion management was established for OAR, especially in extensive aneurysms (i.e., Crawford type I and II aneurysms) [6]. Our data showed that an HLM was used in 70.2% of OAR cases. In comparable data from Germany, the use of an HLM in OAR was substantially lower at 52% [10]. In a clinical study by Coselli et al., an HLM was used in OAR in 45% of cases. Higher rates of HLM usage were reported for Crawford type I and type II aneurysms (60.9%, 82%) compared with the less extensive Crawford types III–V [6]. Compared with clinical data where the Crawford classification and aneurysm diameter are generally available, administrative data is based on the ICD classification that differentiates between abdominal, thoracic, and thoracoabdominal aneurysms only. Therefore, the observed differences may be explained by variations in the data source. Likewise, there were also relevant differences in the use of CSF drainage in OAR between the German data (10%), our Swiss data (6%), and the Coselli data (45%). Due to the structure of administrative data, it is not possible to differentiate between not used and used but not coded. Hence, it must be assumed that a substantial proportion of cases actually received CSF but they were not coded as such.

In our study, renal failure requiring dialysis occurred in 6% after f/bEVAR, 8.7% after OAR, and 20.3% after hybrid procedures for nrTAAA. These findings are comparable with data from a systemic review by Rocha et al. showing 6.4% renal failure after f/bEVAR and 12% after OAR [9]. Further, a more recently published multicentre study from Canada showed 6.9% renal failure after f/bEVAR and 12.5% after OAR [19]. On the other hand, nationwide data from Germany showed a similar trend with lower incidence after f/bEVAR, but substantially higher renal failure rates for all treatment modalities with 10.5% for f/bEVAR, 31.6% for OAR, and 33.5% for hybrid repair [10]. It is noteworthy that the data do not distinguish between acute but reversible renal failure requiring dialysis and permanent renal failure.

Ischaemic complications leading to bowel resection were less frequently observed. We observed a combined overall rate of large and small bowel resection in 2.9% of patients after treatment of nrTAAA. Likewise, bowel resection was more often required in patients treated with OAR that in patients treated with f/bEVAR.

The occurrence of ischaemia of the spinal cord leading to paraplegia is probably the most serious complication, which considerably limits the quality of life. Historic data by Greenberg et al. reported a frequency of 4.3% for endovascular procedures vs. 7.5% for OAR in the elective setting [20]. Our study showed an overall rate of acute paraplegia of 3.9% in the elective setting with lower rates after f/bEVAR (2.6%) compared with OAR (5.2%). After emergency repair, these rates were substantially higher, with 5% after f/bEVAR and 10% after OAR. The higher rate of spinal cord ischaemia in patients with rTAAA compared with nrTAAA may be related to the higher rate of mass transfusions as a surrogate for more severe anaemia and a lower rate of MEPs monitoring and CSF drainage as potentially protective measures. In addition, the concept of a staged endovascular repair to condition the collaterals for spinal cord perfusion is not possible in the emergency setting.

It should be noted that this study only reported on in-hospital mortality and morbidity. A major disadvantage of endovascular therapy is the increased reintervention rate and long-term complications compared with OAR, which is not reflected by this data. This problem is not only known for endovascular therapy of TAAA but also for AAA and endovascular treatment of the aortic arch [21,22]. In the treatment of AAA, late complications after endovascular repair lead to better long-term survival for open surgery after an initial benefit of endovascular therapy [21,23].

### 4.4. Service Providers

The differences between German mortality rates and Swiss mortality rates in the elective setting might be due to the high degree of centralisation for TAAA treatment in Switzerland. Only around 5% of nrTAAA and 8.1% of patients with rTAAA were treated in smaller hospitals in Switzerland. In our multivariable analysis, there was a tendency towards lower mortality in patients who received interhospital transport preoperatively. A previous study had already demonstrated that mortality rates for the treatment of AAA are lower in larger hospitals in Switzerland, and the same results were also demonstrated in Germany [11,24]. In Switzerland, the vast majority of patients with nrTAAA (95%) and rTAAA (91.9%) received treatment at a university or other major hospital compared with only 81.9% in Germany [10] to achieve such a high rate of centralisation, 56% of all patients with rTAAA were transferred prior to surgical treatment. To ensure these prompt transfers, the availability of a 24/7 helicopter emergency medical service is crucial [25].

### 4.5. Limitations

This study had several limitations. First, it was based on administrative data using ICD and CHOP codes, which had the advantage of nearly complete coverage of the Swiss population and a lower risk of selection and information bias for hard endpoints, such as mortality, compared with registry data. However, the lack of individual clinical data and the coding of individual patient identifiers made it difficult to exclude coding errors and allowed for double counting of the same person, leading to incidence bias when patients were treated twice for the same ICD code. The proportion of such patients is unknown but is likely to be small.

Second, the ICD codes do not provide information on the extent of the aneurysm (i.e., no Crawford classification) and do not allow coding of symptomatic patients. The ICD codes only distinguish between abdominal, thoracic, and thoracoabdominal aneurysms. The extent of the aneurysm is an important factor that reflects the disease burden and affects the outcomes. Unreported differences in the extent of aneurysms may partially explain the heterogeneous mortality rates in the literature. Further, the extent of endovascular treatments into the thoracoabdominal segment for juxta- and pararenal aneurysms might lead to an erroneous “upgrading” of abdominal aneurysms–thoracoabdominal aneurysms. This might inflate the incidence of endovascularly treated TAAA. This might lead to an underestimation of complications in the f/bEVAR group, as it might contain invasive complex abdominal aortic aneurysms. A recently published Korean study on ICD coding of ruptured abdominal aortic aneurysms (rAAA) showed that the ICD coding in hospital claims data substantially overestimated the true incidence of rAAA [26]. However, the devastatingly high mortality rate of 97% in patients with rTAAA who were not treated surgically speaks indirectly for the correctness of the diagnosis.

Third, comparisons of mortality rates with international data are limited since adjustments are only possible for age, sex, and Elixhauser score, which do not consider the cardiovascular risk profile or functional capacity of treated individuals.

Finally, in many cases, the administrative data do not allow for a distinction to be made between pre-existing conditions and post-operative complications. Therefore, the distinction between “comorbidity” and “complication” may be erroneous if the acute complications coded were coexisting comorbidities instead.

## 5. Conclusions

Surgical treatment for thoracoabdominal aortic aneurysms dramatically changed from 2009–2018 in Switzerland. The use of endovascular strategies almost tripled in the observed period for nrTAAA and doubled for rTAAA. Endovascular therapy was associated with lower complication rates but not with lower hospital mortality, while aneurysm rupture and a higher Elixhauser comorbidity score were associated with increased hospital mortality.

## Figures and Tables

**Table 1:** Baseline characteristics for the non-ruptured TAAA cohort.

	f/bEVAR *N* = 348	OAR *N* = 309	Hybrid *N* = 79	Total *N* = 736
Age, years	72.2 (7.7)	64.5 (11.2)	64.5 (11.9)	68.2 (10.5)
Male sex	247 (71.0)	214 (69.3)	49 (62.0)	510 (69.3)
Elixhauser score	6 (2–13)	9.5 (2–18)	18 (13–27)	8 (2–17)
Hypertension	257 (73.9)	231 (74.8)	59 (74.7)	547 (74.3)
Myocardial infarction	22 (6.3)	14 (4.5)	6 (7.6)	42 (5.7)
COPD	64 (18.4)	66 (21.4)	18 (22.8)	148 (20.1)
Renal failure	107 (30.7)	63 (20.4)	26 (32.9)	196 (26.6)
PAD	72 (20.7)	74 (23.9)	31 (39.2)	177 (24.0)
Diabetes	44 (12.6)	18 (5.8)	8 (10.1)	70 (9.5)
Type of hospital				
University hospital	286 (82.2)	253 (81.9)	76 (96.2)	615 (83.6)
Non-university hospital	62 (17.8%)	56 (18.1%)	3 (3.8%)	121 (16.4%)
Type of admission				
Direct admission	319 (91.7%)	275 (89.0%)	67 (84.8%)	661 (89.8%)
Transfer from hospital	29 (8.3%)	34 (11.0%)	12 (15.2%)	75 (10.2%)

Data were complete. Counts are presented with percentages in parentheses. Continuous variables are presented as the mean and standard deviation or the median and quartiles (Q1–Q3). TAAA—thoracoabdominal aortic aneurysm; OAR—open aortic repair; f/bEVAR—fenestrated/branched endovascular aortic repair; COPD—chronic obstructive pulmonary disease; PAD—peripheral artery disease.

**Table 2:** Baseline characteristics for the ruptured TAAA cohort.

	f/bEVAR *N* = 43	OAR *N* = 41	Hybrid *N* = 8	None *N* = 57	Total *N* = 149
Age, years	74.8 (8.8)	67.4 (13.2)	68.4 (10.1)	81.7 (8.6)	75.1 (11.7)
Male sex	26 (60.5)	26 (63.4)	8 (100)	37 (64.9)	97 (65.1)
Elixhauser score	10 (5–21)	15 (8–21)	5 (1–19)	3 (0–6)	8 (2–17)
Hypertension	34 (79.1)	19 (46.3)	5 (62.5)	18 (31.6)	76 (51.0)
Myocardial infarction	2 (4.7)	5 (12.2)	0 (0.0)	1 (1.8)	8 (5.4)
COPD	4 (9.3)	6 (14.6)	1 (12.5)	6 (10.5)	17 (11.4)
Renal failure	14 (32.6)	14 (34.1)	3 (37.5)	12 (21.1)	43 (28.9)
PAD	11 (25.6)	7 (17.1)	4 (50.0)	4 (7.0)	26 (17.4)
Diabetes	4 (9.3)	2 (4.9)	2 (25.0)	1 (1.8)	9 (6.0)
Type of hospital					
University hospital	35 (81.4%)	33 (80.5%)	8 (100.0%)	20 (35.1%)	96 (64.4%)
Non-university hospital	8 (18.6%)	8 (19.5%)	0 (0.0%)	37 (64.9%)	53 (35.6%)
Type of admission					
Direct admission	15 (34.9%)	18 (43.9%)	5 (62.5%)	47 (82.5%)	85 (57.0%)
Transfer from hospital	28 (65.1%)	23 (56.1%)	3 (37.5%)	10 (17.5%)	64 (43.0%)

Data were complete. Counts are presented with percentages in parentheses. Continuous variables are presented as the mean and standard deviation or the median and quartiles (Q1–Q3). TAAA—thoracoabdominal aortic aneurysm; OAR—open aortic repair; f/bEVAR—fenestrated/branched endovascular aortic repair; COPD—chronic obstructive pulmonary disease; PAD—peripheral artery disease.

**Table 3:** Management and outcomes in the non-ruptured TAAA cohort.

	f/bEVAR *N* = 348	OAR *N* = 309	Hybrid *N* = 79	Total *N* = 736
Procedural management				
HLM	6 (1.7)	217 (70.2)	49 (62.0)	272 (37.0)
ECMO	1 (0.3)	4 (1.3)	1 (1.3)	6 (0.8)
CSF drainage	7 (2.0)	18 (5.8)	37 (46.8)	62 (8.4)
MEPs monitoring	4 (1.1)	65 (21.0)	43 (54.4)	112 (15.2)
Transfusion management				
Autotransfusion	14 (4.0)	180 (58.3)	57 (72.2)	251 (34.1)
Packed red blood cells				
1–5	84 (24.1)	74 (23.9)	26 (32.9)	184 (25.0)
>5	43 (12.4)	99 (32.0)	41 (51.9)	183 (24.9)
Fresh frozen plasma				
1–5	6 (1.7)	11 (3.6)	10 (12.7)	27 (3.7)
>5	1 (0.3)	12 (3.9)	21 (26.6)	34 (4.6)
Platelet concentrate				
1–5	5 (1.4)	17 (5.5)	11 (13.9)	33 (4.5)
>5	1 (0.3)	4 (1.3)	5 (6.3)	10 (1.4)
Complications				
Large bowel resection	5 (1.4)	9 (2.9)	2 (2.5)	16 (2.2)
Small bowel resection	1 (0.3)	4 (1.3)	0 (0.0)	5 (0.7)
Lower limb fasciotomy	1 (0.3)	1 (0.3)	0 (0.0)	2 (0.3)
CVVHD	21 (6.0)	27 (8.7)	16 (20.3)	64 (8.7)
Major amputation	0 (0.0)	0 (0.0)	1 (1.3)	1 (0.1)
Acute paraplegia	9 (2.6)	16 (5.2)	4 (5.1)	29 (3.9)
Outcomes				
ICU stay, hours	0 (0–46)	48 (20–123)	43 (18–157)	24 (0–90)
Hospital stay, days	8 (5–16)	15 (11–22)	16 (13–23)	13 (8–20)
Mortality	23 (6.6)	23 (7.4)	6 (7.6)	52 (7.1)

Data were complete. Counts are presented with percentages in parentheses. Continuous variables are summarised with median and quartiles (Q1–Q3). HLM—heart–lung machine; ECMO—extracorporeal membrane oxygenation; CSF—cerebrospinal fluid drainage; MEPs—motor evoked potentials; CVVHD—continuous veno-venous haemodialysis; ICU—intensive care unit.

**Table 4:** Management and outcomes in the ruptured TAAA cohort.

	f/bEVAR *N* = 43	OAR *N* = 41	Hybrid *N* = 8	None *N* = 57	Total *N* = 149
Procedural management					
HLM	1 (2)	27 (66)	3 (38)	0	31 (21)
ECMO	0	2 (5)	0	0	2 (1)
CSF drainage	0	1 (2)	1 (13)	0	2 (1)
MEPs monitoring	0	2 (5)	1 (13)	0	3 (2)
Transfusion management					
Autotransfusion	0	17 (42)	7 (88)	1 (1.8)	25 (17)
Packed red blood cells					
1–5	15 (35)	5 (12)	3 (38)	6 (10.5)	29 (20)
>5	15 (35)	17 (42)	3 (38)	4 (7.0)	39 (26)
Fresh frozen plasma					
1–5	0	1 (2)	1 (13)	0	2 (1)
>5	3 (7)	2 (5)	1 (13)	0	6 (4)
Platelet concentrate					
1–5	2 (5)	1 (2)	1 (13)	0	4 (3)
>5	1 (2)	0	0	0	1 (1)
Complications					
Large bowel resection	3 (7)	2 (5)	0	0	5 (3)
Small bowel resection	1 (2)	1 (2)	0	0	2 (1)
Lower limb fasciotomy	0	0	0	0	0
CVVHD	6 (14)	5 (12)	0	0	11 (7)
Major amputation	0	0	0	0	0
Acute paraplegia	2 (5)	4 (10)	0	0	6 (4)
Outcomes					
ICU stay, hours	62 (26–234)	43 (15–213)	22 (13–81)	0 (0–0)	14 (0–72)
Hospital stay, days	16 (7–30)	13 (4–23)	14 (8–20)	1 (1–1)	3 (1–18)
Mortality	12 (28)	14 (34)	1 (13)	55 (97)	82 (55)

Data were complete. Counts are presented with percentages in parentheses. Continuous variables are sumarised using the median and quartiles (Q1–Q3). HLM: heart–lung machine; ECMO: extracorporeal membrane oxygenation; CSF: cerebrospinal fluid drainage; MEPs: motor evoked potentials; CVVHD: continuous veno-venous haemodialysis; ICU: intensive care unit.

## Data Availability

The full dataset can be requested from the Federal Statistical Office of Switzerland, Espace de l’Europe 10, CH-2010 Neuchâtel, Switzerland.

## Supplementary Materials

The following supporting information can be downloaded at https://www.mdpi.com/article/10.3390/jcm12165213/s1, Table S1: Baseline Characteristics, Procedural Details and Outcomes for nrTAAA by Time Period; Table S2: Baseline Characteristics, Procedural Details and Outcomes for rTAAA by Time Period.
