# Supplementary material for: Hospital Incidence, Treatment, and Outcome of 885 Patients with Thoracoabdominal Aortic Aneurysms Treated in Switzerland over 10 Years—A Secondary Analysis of Swiss DRG Data
Source: J Clin Med. 2023 Aug 10;12(16):5213. doi: 10.3390/jcm12165213 (PMC10455290; doi:10.3390/jcm12165213)
Supplement: Supplementary file 1 [file jcm-12-05213-s001.zip › jcm-2514171-supplementary.pdf]

Journal of
Clinical Medicine

Table S1. Baseline Characteristics, Procedural Details and Outcomes for nrTAAA by Time Period.

	2009–2013 (*N* = 274)	2014–2018 (*N* = 462)	*p* Value
Baseline Characteristics			
Age, years	67.1 (10.7)	68.7 (10.4)	0.047
Male sex	193 (70.4%)	317 (68.6%)	0.604
van Walraven Score	5 (2–13)	11 (3–18)	< 0.001
Hypertension	202 (73.7%)	345 (74.7%)	0.775
Myocardial Infarction	7 (2.6%)	35 (7.6%)	0.005
COPD	55 (20.1%)	93 (20.1%)	0.985
Renal Failure	57 (20.8%)	139 (30.1%)	0.006
PAD	58 (21.2%)	119 (25.8%)	0.159
Diabetes	252 (92.0%)	414 (89.6%)	0.291
Type of Hospital			0.992
University Hospital	229 (83.6%)	386 (83.5%)	
Non-University	45 (16.4%)	76 (16.5%)	
Type of Admission			0.323
Direct Admission	250 (91.2%)	411 (89.0%)	
Transfer from Hospital	24 (8.8%)	51 (11.0%)	
Procedural Management			
HLM	131 (47.8%)	141 (30.5%)	< 0.001
ECMO	0 (0.0%)	6 (1.3%)	0.058
CSF Drainage	0 (0.0%)	62 (13.4%)	< 0.001
MEPs Monitoring	37 (13.5%)	75 (16.2%)	0.319
Transfusion Management			
Autotransfusion	93 (33.9%)	158 (34.2%)	0.943
Packed red blood cells			< 0.001
1–5	52 (19.0%)	132 (28.6%)	
>5	46 (16.8%)	137 (29.7%)	
Fresh Frozen Plasma			< 0.001
1–5	0 (0.0%)	27 (5.8%)	
>5	0 (0.0%)	34 (7.4%)	
Platelet concentrate			< 0.001
1–5	0 (0.0%)	33 (7.1%)	
>5	0 (0.0%)	10 (2.2%)	
Complications			
Large Bowel Resection	8 (2.9%)	8 (1.7%)	0.285
Small Bowel Resection	3 (1.1%)	2 (0.4%)	0.291
Lower Limb Fasciotomy	1 (0.4%)	1 (0.2%)	0.708
CVVHD	18 (6.6%)	46 (10.0%)	0.115
Major Amputation	1 (0.4%)	0 (0.0%)	0.194
Acute Paraplegia	13 (4.7%)	16 (3.5%)	0.388
Outcomes			
ICU Stay, hours	25 (0–101)	24 (0–84)	0.220
Hospital Stay, days	13 (90–22)	12 (7–19)	0.035
Mortality	19 (6.9%)	33 (7.1%)	0.915

Table S2. Baseline Characteristics, Procedural Details and Outcomes for rTAAA by Time Period.

	2009–2013 (N = 62)	2014–2018 (N = 87)	p Value
Baseline Characteristics			
Age, years	74.6 (12.7)	75.4 (11.0)	0.67
Male sex	42 (67.7%)	55 (63.2%)	0.568
van Walraven Score	8 (3 to17)	8 (0–17)	0.619
Hypertension	31 (50.0%)	45 (51.7%)	0.836
Myocardial Infarction	2 (3.2%)	6 (6.9%)	0.327
COPD	8 (12.9%)	9 (10.3%)	0.628
Renal Failure	18 (29.0%)	25 (28.7%)	0.969
PAD	12 (19.4%)	14 (16.1%)	0.605
Diabetes	3 (4.8%)	6 (6.9%)	0.603
Type of Hospital			0.715
University Hospital	41 (66.1%)	55 (63.2%)	
Non-University	21 (33.9%)	32 (36.8%)	
Type of Admission			0.901
Direct Admission	35 (56.5%)	50 (57.5%)	
Transfer from Hospital	27 (43.5%)	37 (42.5%)	
Procedural Management			
HLM	18 (29.0%)	13 (14.9%)	0.037
ECMO	0 (0.0%)	2 (2.3%)	0.229
CSF Drainage	1 (1.6%)	1 (1.1%)	0.809
MEPs Monitoring	1 (1.6%)	2 (2.3%)	0.769
Transfusion Management			
Autotransfusion	14 (22.6%)	11 (12.6%)	0.11
Packed red blood cells			< 0.001
1–5	5 (8.1%)	24 (27.6%)	
>5	11 (17.7%)	28 (32.2%)	
Fresh Frozen Plasma			0.049
1–5	0 (0.0%)	2 (2.3%)	
>5	0 (0.0%)	6 (6.9%)	
Platelet concentrate			0.158
1–5	0 (0.0%)	4 (4.6%)	
>5	0 (0.0%)	1 (1.1%)	
Complications			
Large Bowel Resection	2 (3.2%)	3 (3.4%)	0.941
Small Bowel Resection	0 (0.0%)	2 (2.3%)	0.229
Lower Limb Fasciotomy	0 (0.0%)	0 (0.0%)	NA
CVVHD	4 (6.5%)	7 (8.0%)	0.714
Major Amputation	0 (0.0%)	0 (0.0%)	NA
Acute Paraplegia	4 (6.5%)	2 (2.3%)	0.204
Outcomes			
ICU Stay, hours	11 (0–78)	17 (0–64)	0.677
Hospital Stay, days	3 (1–18)	5 (1–19)	0.692
Mortality	33 (53.2%)	49 (56.3%)	0.708
